# Design of Cultured Neuron Networks *in vitro* with Predefined Connectivity Using Asymmetric Microfluidic Channels

**DOI:** 10.1038/s41598-017-15506-2

**Published:** 2017-11-15

**Authors:** Arseniy Gladkov, Yana Pigareva, Daria Kutyina, Vladimir Kolpakov, Anton Bukatin, Irina Mukhina, Victor Kazantsev, Alexey Pimashkin

**Affiliations:** 1 Lobachevsky State University of Nizhny Novgorod, Laboratory of Neuroengineering, Nizhny Novgorod, 603950 Russia; 2 Nizhny Novgorod State Medical Academy, Central Research Laboratory, Nizhny Novgorod, 603950 Russia; 3 Saint-Petersburg National Research Academic University of the RAS, Laboratory of Nanobiotechnology, Saint-Petersburg, 194021 Russia

## Abstract

The architecture of neuron connectivity in brain networks is one of the basic mechanisms by which to organize and sustain a particular function of the brain circuitry. There are areas of the brain composed of well-organized layers of neurons connected by unidirectional synaptic connections (e.g., cortex, hippocampus). Re-engineering of the neural circuits with such a heterogeneous network structure in culture may uncover basic mechanisms of emergent information functions of these circuits. In this study, we present such a model designed with two subpopulations of primary hippocampal neurons (E18) with directed connectivity grown in a microfluidic device with asymmetric channels. We analysed and compared neurite growth in the microchannels with various shapes that promoted growth dominantly in one direction. We found an optimal geometric shape features of the microchannels in which the axons coupled two chambers with the neurons. The axons grew in the promoted direction and formed predefined connections during the first 6 days *in vitro* (DIV). The microfluidic devices were coupled with microelectrode arrays (MEAs) to confirm unidirectional spiking pattern propagation through the microchannels between two compartments. We found that, during culture development, the defined morphological and functional connectivity formed and was maintained for up to 25 DIV.

## Introduction

The development of bioengineering in recent years permits the design of neuronal cell cultures with a defined network architecture that addresses many fundamental questions such as cell to cell interactions^1,2^, axon isolation and guidance to study brain development^3^ and neural degeneration and damage^4,5^. Reconstruction of brain circuitry *in vitro* can be used to study basic mechanisms of information processing and specific molecular pathways in the brain^2,6,7^. Directed synaptic pathways that provide signal transfer are essential in the hippocampus, cortical columns and other brain areas. Similar directed connectivity in artificial neural circuits *in vitro* can be organized by the guidance of neurites between isolated groups of cells^8–10^. Several techniques were proposed for the manipulation of axon growth in neuronal cultures, including micropatterning with microcontact printing of adhesive proteins^11–14^, axon growth through microchannels using microfluidics^8,9,15–19^, the constant flow of culture medium^20^, high-frequency electrical field application to the axon in the microchannel and collagen scaffolds^21,22^, the creation of gradients of trophic^23^ and growth factors^24^ or extracellular matrix^25^, and the modification of surface microtopography with miсro and nano grooves or pillars^26–28^.

The method of directed connectivity construction with micropatterning technology^13^ is based on the finding that boundaries of adhesive geometric structures affect neurite outgrowth^29^. The direction of the neurite growth can be controlled by a specific triangular design of the boundaries^11,14,30^. The triangular shape forms a narrow bottleneck that provides directional growth of the axons and spiking activity propagation alongside the growth of connectivity. The triangle-shaped micropatterns with concave sides were associated with more efficient desired axon guiding^11,31^. It was shown that the number of triangular *micropatterns* in their sequence correlated with the efficiency of the neural network to propagate Ca^2+^ bursts in one direction^30^. However, such a method does not permit the separation of cell bodies and neurites in the micropatterns. The neurites can grow on top of other neurites and form multilayers because there is no limitation in the vertical plane, and the precise localization of the axons and synapses cannot be predefined.

The other approach for shaping neural network architecture was based on microfluidic methods. The microfluidic approach permitted the cells to be plated in small chambers connected by microchannels through which axons grew for several days and were synaptically coupled to the chambers. The microchannel width and height could be smaller than the cell somata size, which ensures the precise isolation of the cellular population and the axons.

Several approaches exist for the construction of the unidirectional connection in neuronal networks. One approach consisted of first plating the cells into the *Source* chamber, then plating them into the *Target* chamber. First, the cells were plated in the *Source* chamber, and the axons grew through the microchannels to the empty *Target* chamber over one week. Next, new cells were plated into the *Target* chamber, which was already filled with the axons. This approach permitted the formation of synapses only in the *Target* chamber and promoted one-way connectivity development^15^. The dominant growth of the cortical axons in the promoted direction may be provided by special traps (barriers) in the microchannels^9^ or the funnel-shape of the channel^2,6,10^. It was shown that an axon overcame only two traps when it grew in *the opposite of the desired direction* ^9^. However, microchannels with multiple bottlenecks and various shapes were not studied and may provide more efficient unidirectional growth due to the limitation of *opposite* growth.

Spiking activity in cultured neural networks is characterized by spontaneously generated signals in the form of synchronized network burst discharges^32–37^. The microfluidic chips can be combined with microelectrode array (MEA) to record electrical activity in developing culture networks. MEA can effectively detect spike propagation in growing axons through the microchannels^9,15,38–40^. Such a microfluidic approach can be expanded to engineer multiple clusters of various cell types connected by unidirectional pathways with long-term electrical activity recording and stimulation. In this context, it may be interesting to develop a simple and robust method to simultaneously plate many cellular clusters separately and guide axon growth between them.

In this study, we presented a technique for the creation of neural circuits *in vitro* with unidirectional communication between two subpopulations of primary hippocampal neurons (E18) in a microfluidic device. We studied neurite growth in microchannels with various asymmetric shapes that promote growth dominantly in one direction. We found the optimal geometry of the microchannels that permitted the one-way synaptic connectivity of two cell chambers. The axons grew in *the promoted direction* and formed *predefined connections* between two neuronal subpopulations for the first 6–10 days *in vitro* (DIV). The microfluidic chip was integrated with MEA substrate, and spike propagation between the two compartments was monitored. We also found that during culture development, the unidirectional connectivity was effectively preserved for up to 25 DIV.

## Methods

### Microfluidic device fabrication

The microfluidic chips were fabricated via polydimethylsiloxane (PDMS) moulding techniques. Standard two-layer lithography was used for mould fabrication. For details on this process, see Malyshev E. *et al*., 2015^16^. The surfaces of the prepared PDMS chips were mounted with microelectrode arrays (MEAs) and glasses, which were coated with the adhesion promoting molecule polyethyleneimine at a concentration of 1 mg/mL (Sigma-Aldrich, P3143, USA).

**Figure 1:**
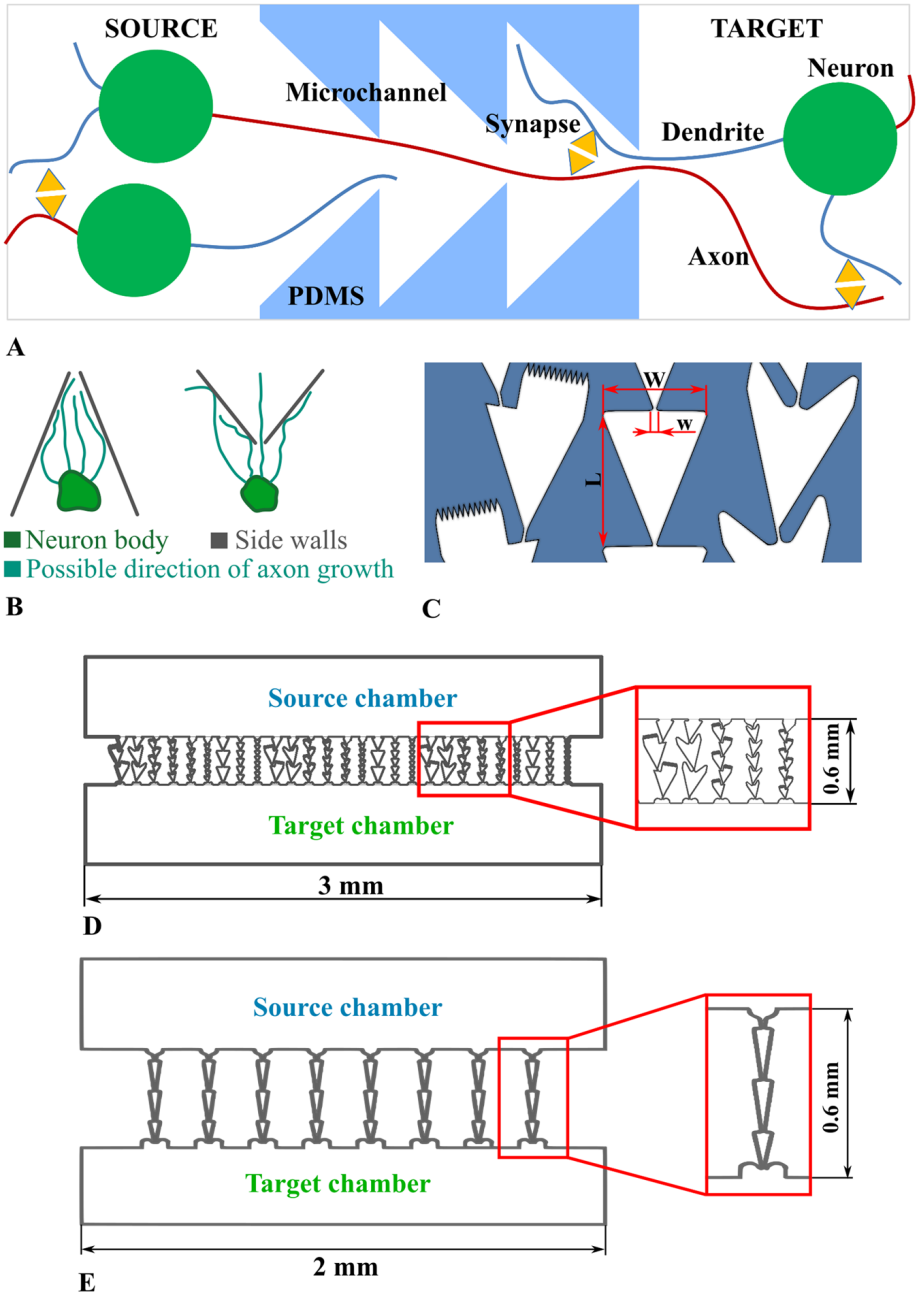
Microfluidic chip with microchannels for unidirectional axon growth between two cultured networks. (**A**) Schematic view of neural network connectivity in the device with two chambers and microchannels. The shape of the microchannel provides axon growth from the *Source* to the *Target* chamber. (**B**) Schematic view of axon growth through bottlenecks (**C**) Shapes: “Spines”, “Triangles” and “Zig-zag”. Characteristic dimensions: Width (W) = 60–160 µm, Bottleneck width (w) = 5 µm, Height = 5 µm, Length (L) = 66, 70, 100, 200 µm. (**D**) Microfluidic chip with 9 variations of 3 types of microchannels. (**E**) Microfluidic chip with triangle shaped microchannels. Bottleneck width (w) = 7 µm, Width (W) = 60 µm, Length (L) = 200 µm.

According to the predefined direction of axon growth, the subnetworks were labelled *Source* and *Target* chambers. We defined as *Start* and *End* the joints of the microchannel with the *Source* and the *Target* chamber, respectively. The microchannel consisted of a sequence of segments. The design of the segment’s shape facilitated the directed axon growth to the *Target* chamber due to convergent walls. Such a shape reduced the probability of backward growth by subsequent bottleneck and special traps. We studied three types of segments as follows: “Zig-zag”, “Spines” and “Triangle” (Fig. 1C) with three different lengths of 70 µm (66 µm for “Triangle”), 100 µm and 200 µm; and segment widths of 60, 80 and 160 µm, respectively. The “bottleneck” diameter was 5 µm thick, and the tip of each shape had a 45° guiding corner. “Zig-zag” and “Spines” types had traps for axons growing from the *Target* to the *Source* chamber. We also evaluated microchannels with narrower “Triangle” segments of 200 µm length, 40 µm width and 7 µm thickness with a “bottleneck” diameter (Fig. 1E). The tip of the guiding corner was 10°.

**Figure 2:**
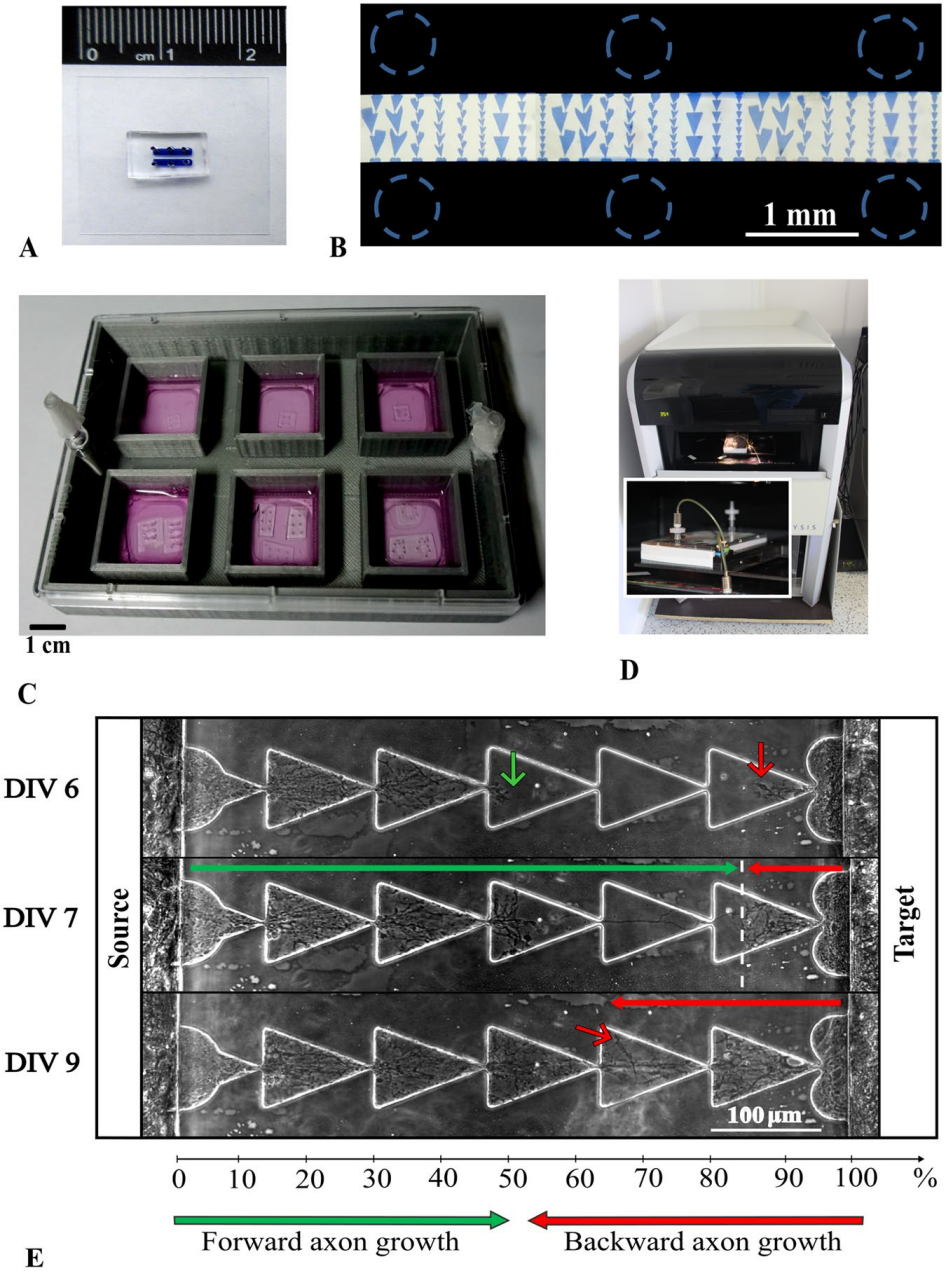
Experimental setup to study neurite growth in microfluidic chips. (**A**) Two-chamber microfluidic chip (**B**) Photo of the chip filled with methylene blue; dashed circles marked punch holes for cell plating. Bar length 1 mm (**C**) A 6-well plate with microfluidic chips attached to coverslips. Bar length 1 cm. (**D**) The automated inverted microscope system Cell IQ (see Methods) was used to study neurite growth from the 6-well plate (see Methods). (**E**) Example of neurite outgrowth during development: top- DIV 6, middle- DIV 7 when axons from the *Source* chamber met oppositely grown axons from the *Target* chamber, bottom- DIV 9 when the axon grew from *Target* chamber alongside the neurons from the *Source* chamber, and both were still distinguishable (see Support Video S6).

To study neurite growth in the microchannels, we fabricated multi-well plates (Fig. 2C). A model of the multi-well plate with holes on the bottom for coverslips was 3D printed with polylactide (PLA) plastic ((Ultimaker, Netherlands) 2.85 mm diameter, UM-9015-A). Coverslips at a size of 24 mm were glued to the bottom of the PLA skeleton with liquid PDMS (10:1) and cured in an oven at 70 °C for four hours.

PDMS chips for multi-well plates were punched by a biopsy puncher (Kai medical, Germany) 0.5 mm in diameter. Two holes were made in the opposite outer corners for each chamber. Each PDMS chip was positioned and mounted onto the clear surface of coverslips using oxygen plasma (Plasma system FEMTO, Diener, Germany) that activated the glass and PDMS surfaces. The power was set to 100 W, and oxygen flow was 5 sm^3^/min. After exposure for 1 minute, the plasma system was turned off and oxygen was replaced by nitrogen. Then, the PDMS chips were quickly mounted onto the glasses and cured in an oven at 100 °C for 30 min.

To study the electrical activity of the cultures, we coupled two types of PDMS chips with “Zig-zag” segments (length 100 µm) and narrow “Triangle” segments of the microchannels with MEA substrates. We punched open reservoir structures with a binocular and a rectangular blade puncher (l~2 mm, w~1.5 mm) in PDMS chips that were made on the chamber area. The chips were manually aligned with the MEA, which was composed of 60 electrodes (TiN electrodes, diameter 30 µm with 200 µm in between, Multichannel Systems, Germany), via a three-dimensional mechanical micromanipulator under a binocular. Furthermore, 14 electrodes were placed in the *Source* chamber, 24 electrodes were placed in the microchannels (3 electrodes in each of 8 microchannels), and 22 electrodes were placed in the *Target* chamber. We used reversible bonding for MEAs to prevent damage of the electrodes. After PDMS chips were mounted to the MEA, it was cured in an oven at 80 °C for 30 min.

### Cell culturing

Hippocampal cells were dissociated from embryonic mice (E18) and plated in the cell chambers of PDMS chips at an initial density of approximately 7,000–9,000 cells/mm^2^. Mice were euthanized via cervical dislocation according to protocols approved by the National Ministry of Health for the care and use of laboratory animals. The protocol was approved by the Committee on the Ethics of Animal Experiments of the Nizhny Novgorod State Medical Academy. All efforts were made to minimize suffering. For culturing procedure details see Pimashkin *et al*., 2013^39^. The cells were cultured under constant conditions of 35.5 °C, 5% CO_2_ in a humidified cell culture incubator (MCO-18AIC, SANYO, Japan).

### Imaging

Neurite outgrowth dynamics were analysed with a commercial system for the continuous monitoring of living cells in culture and image analysis (Cell IQ, ChipMan Technologies, Finland). The automatic system was used to monitor morphological dynamics of several cultures in the multi-well plates simultaneously in different regions. We started to monitor neurite growth 24 h after plating at the first medium change. The observation ended after 10 days of network development when all microchannels were usually filled with neurites. The Cell IQ system continuously acquired phase contrast images with a ×20 objective (Nikon CFI Plan Fluorescence ELWD ADL, Japan) at an interval of 20 min for each selected region. Data were processed and analysed with a Cell IQ Analyzer program and ImageJ.

To quantify the efficiency of the microchannel in guiding neurite growth in one direction from the *Source* culture to the *Target* culture, we defined a *Forward/backward meeting point* for neurites grown from the *Source* and the *Target* cultures (Fig. 2E, middle panel, dashed line). *Forward/backward meeting point* was measured as the distance in which the neurite from the *Target* chamber grew through the microchannel until it crossed the neurites growing from the *Source* chamber. The distance was estimated relative to the channel length and was presented as a percentage. Then, the neurites grew further alongside the crossed neurites until the shape of the neurites could no longer be distinguished. At that moment, we measured the distance of the neurite growth from the *Target* chamber, which was defined as the *Maximum backward neurite growth* (Fig. 2E, bottom panel, red arrow). If the neurite grew from the *Source* chamber and reached the *Target* chamber while no axons grew from the *Target* chamber, then the defined *Forward/backward meeting* would be equal to 0%. *Forward/backward growth velocity* was calculated as the velocity of one neurite while it passed two bottlenecks (the length of microchannel segment was divided by the time that the neurite spent to pass the segment).

### Immunostaining

For immunostaining, we used cultures grown in microfluidic chips bonded to the glasses. The chips were mounted via a reversible method to remove the chips from glasses to access the neurites in the microchannels for staining. On 6 DIV, the neuronal cultures were taken for immunostaining. Culture medium was first washed with warm (37 °C) PBS. Then, cells were fixed with warm (37 °C) freshly prepared 4% paraformaldehyde (Sigma-Aldrich, USA) for 15 min at room temperature and then washed with PBS three times for 5 min. The microfluidic chamber was removed to access the neurites in the microchannels for immunohistochemistry (ICC) staining. Cells were permeabilized with 0.1% Triton X-100 in PBS with 2% BSA (Bovine Serum Albumin) for 20 min. To assess the expression of neurons and axons, antibodies against Guinea pig β3-tubulin (SYSY, 302 304, Germany) and mouse tau (SYSY, 314 011, Germany), respectively, were used. Cells were incubated with primary antibodies at room temperature for 2 h and then washed with PBS buffer three times for 5 min. Goat anti-Guinea Pig Alexa Fluor 488 and Goat anti-Mouse Alexa Fluor 647 secondary antibodies (Thermo Fisher Scientific, USA) were used for β3-tubulin and tau, respectively. Cells were incubated in the dark with secondary antibodies at room temperature for 30 min and then washed with PBS and with deionized water to remove salt. After that, cells were fixed in mounting medium (Sigma-Aldrich, USA) and imaged with a confocal microscope (Zeiss LSM 710, Germany). Four-layer z-stacks reached 5 µm. Images were taken with a 3.15 µm pinhole. Then, stack images were averaged separately for each channel in ImageJ.

### Electrophysiology

Spiking activity was recorded from 59 (1 reference) TiN electrodes of the MEA system (Multichannel Systems, Germany) at a sample rate of 20 kHz. Stimulation through the MEA was performed with the STG-4004 stimulator (Multichannel Systems, Germany). Detection of the recorded spikes was based on the threshold calculation of the signal median as described in our previous studies^41–43^. We applied a previously described method to detect and estimate network bursts^41^ of neuronal cultures. All signal analyses and statistics were performed with custom made software in Meaman in Matlab.

### Burst propagation analysis

Spontaneous activity was recorded every 5 days from 10 DIV until 25 DIV. On 10 DIV, two cultures in the microfluidic device were already coupled by the axons through the microchannels and generated spontaneous bursting activity. Some of the bursts in the *Source* chamber evoked bursts in the *Target* chamber with a small delay. First, we detected the bursts in each chamber separately with algorithms described in our previous studies^41^. Then, we calculated burst fractions in the *Target* culture that were evoked by the *Source* culture spontaneous activations and vice-versa.

We found that for each burst in the *Target* culture (*Target* burst), the burst in the *Source* culture (*Source* bursts), if present, satisfied two conditions. First, the interval between the beginning of the two bursts was in the range of −500 ms to 500 ms. Second, the starting point of the latest burst among the two should not be later than the end of the preceding one. In other words, the burst in one chamber was not to be evoked from the other one if there was no spiking activity in between. Thus, we defined the inter-chamber signal propagation as a continuous dynamic process that originated in one chamber then propagated through the axons and caused the response burst while the initial one was still spiking. Next, we tested the hypothesis that the observed delays reflected true burst propagation between the chambers rather than a random appearance of the two bursts by means of surrogate data analysis. The surrogate data were derived from the original sequence of the bursts by jittering burst time occurrence with a random delay with uniform distribution. This shuffling procedure was repeated 1000 times, and on each iteration, the delays between the *Target* and *Source* bursts were collected, as for the original data. Based on the surrogates, we estimated a probability of a random appearance of the delays, e.g., burst propagation between cultures for each delay timebin (Fig. S1, red line). The probability of burst propagation delays from the original data was 5 times higher than the standard deviation of the surrogate data, and hence, these delays were considered physiologically relevant and were used in further analyses. To estimate burst propagation in the forward direction, we measured the number of *Target* bursts that appeared after the *Source* bursts. The number of such propagated bursts relative to the total number of *Source* bursts was defined as the *Source-to-Target Propagation Probability* (PP_S-T_). The percentage of Source bursts followed by *Target* bursts was defined as a *Target-to-Source Propagation Probability* (PP_T-S_). To quantify the microchannel efficiency to propagate the spiking patterns in the desired direction from the *Source* culture to the *Target* culture, we defined a *directional propagation index* (DPI), as the relationship of PP_S-T_ to PP_T-S_. The PP_S-T_, PP_T-S_ and DPI were estimated for each experiment and then averaged over all cultures at 10, 15, 20 or 25 DIV.

Data Availability. The code and datasets generated during and/or analysed during the current study are available from the corresponding author upon reasonable request.

## Results

### Neurite growth in the microchannels

**Figure 3:**
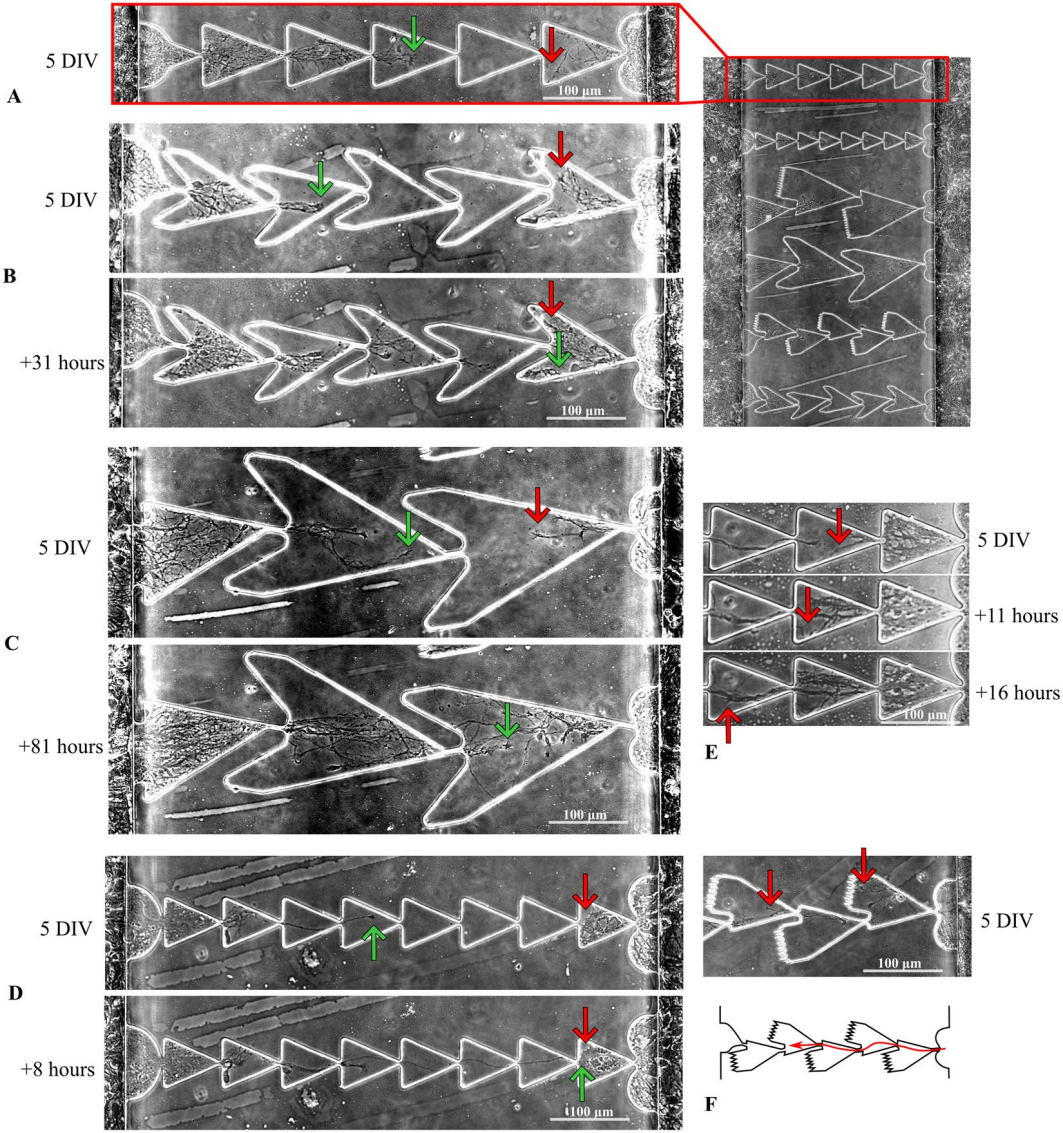
Directional neurite growth in the various shaped microchannels. (**A**) Two neuronal cultures grown on microfluidic chips with asymmetric microchannels guiding the neurites from the *Source* chamber (left) to the *Target* chamber (right). The image was obtained on the 5th day *in vitro* (DIV). (**B**) “Zig-zag” shape of the microchannel composed of medium sized sections (100 µm length). Group of neurites growing dominantly in one direction (left to right, green arrow) through bottlenecks, while opposite direction growth (red arrow) was limited by “horn” traps. On the 7th DIV, the neurites from the *Source* chamber reached the final segment where they met the neurites grown from the *Target*. (Support Video S1). (**C**) Neurite growth in large “Zig-zag” segments (see Methods). Neurites passed 2 bottlenecks and met neurites from the *Target* culture on the 8th DIV. (Support Video S2). (**D**) Neurites in “triangle” shaped microchannels passed 8 bottlenecks and reached the final segment on the 5th DIV, while neurites from the *Target* chamber passed only one and remained in the last segment the whole time. (Support Video S3). (**E**) Neurites growing “backward” from the *Target* culture alongside the neurites grown from the *Source*. (Support Video S4). (**F**) Neurites in “Spine” shaped segments may grow from the *Target* culture into a trap (upper red arrow), while others may grow through the bottleneck alongside the boundary (lower arrow). (Support Video S5).

First, we investigated neurites in the hippocampal culture network that grew through asymmetric microchannels of the microfluidic device (Fig. 1). We plated primary hippocampal neurons (E18) on 10 microfluidic chips attached to a custom designed 6-well plate (See Methods) (Fig. 2C). The cultured neurons, within 48 h after plating, started to release neurites between the cells and into the microchannels simultaneously from the *Source* and *Target* chambers (Fig. 3A). Continuous image acquisition was used to monitor the outgrowth of individual neuronal processes during culture development for up to 10 days (see Methods). We found that the neurites that originated from the *Source* chamber sprouted within the microchannel sections and bottlenecks without any visible resistance. The neurites from the *Target* chamber also entered the microchannel and passed at least one bottleneck (Fig. 3B,C,D). Then, the neurites went alongside the boundaries mostly to the lateral sides of the channel where they met a “trap” structure. In general, most of the neurites from the *Source* chamber grew through the microchannel in the forward direction during the first 2–5 days (Fig. 3B,C,D). In the opposite direction, the axons grew in at least one section. For each example of neurite outgrowth presented in Fig. 3, we prepared a video that contained timelapse images of several days of the observed process (Support Videos S1–S5). The neurites in the “Zig-zag” type sections were caught in the lateral “horn” traps that prevented further growth (Fig. 3B). Interestingly, in some cases, the neurites did not stop or retract, they continued to elongate and seek possible directions and could change the growth angle up to 180°. Triangular sections also showed similar dynamics (Fig. 3D), while in the “Spine” shaped sections, most of the neurites were caught in one lateral trap, but the neurites that grew by the other side could pass the bottleneck (Fig. 3F).

**Figure 4:**
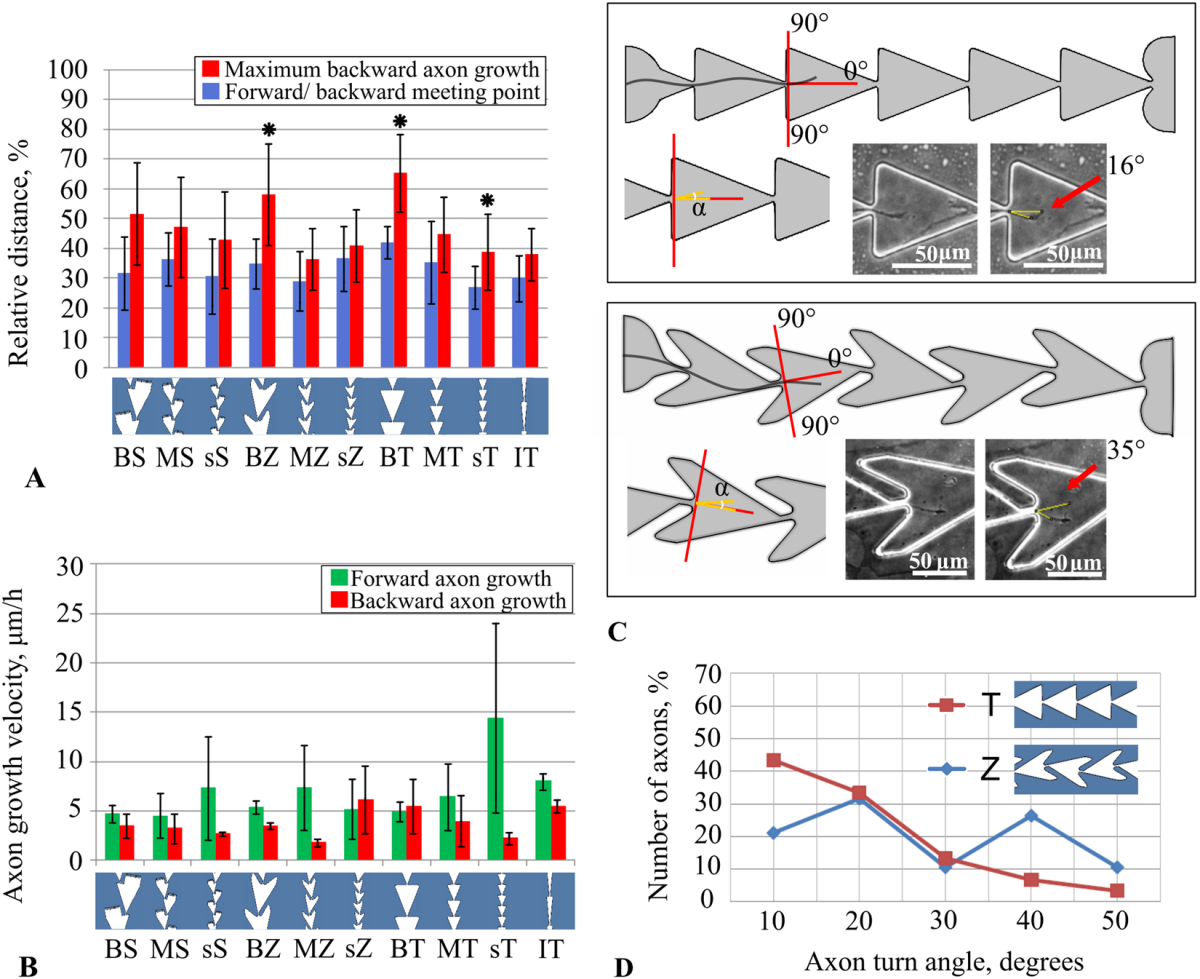
Neurite growth characteristics for various microchannel designs. (**A**) *Forward/backward meeting point* - the point where forward axon growth met backward growth, counting from the end of the channel (blue bars). *Maximum backward neurite growth* was defined as the maximum visible lengths of the processes growing from the *Target* chamber (red bars). The values represented the growth distance relative to microchannel length, which was 600 µm (means ± SD, n = 6 cultures, 138 microchannels). (**B**) Growth velocities for forward and backward directions measured within a single segment (mean ± SD, n = 6 cultures, 121 segments). (**C**) Angle of the neurite growth trajectory when it passed the bottleneck going to the *Target* chamber. (**D**) Distribution of the neurite outgrowth angles from “Triangle” and “Zig-zag” bottleneck types.

Next, we estimated the efficiency of directional neurite growth for all microchannel types. First, for each microchannel, we determined the distance that the axon passed from the *Source* chamber to the point where it met the neurites grown from the *Target* chamber (Fig. 2E, middle). This forward/backward meeting point represented the efficiency of the microchannel to provide unidirectional growth. We analysed the experiments from 6 cultures and 138 microchannels in total. For each channel type, we estimated a mean value and standard deviation of the forward/backward meeting point as a distance measured from the end of the microchannel (Fig. 4A, blue). Note that the least effective channel types were large size segments BT, while the others were relatively similar. However, one-way ANOVA (F-test) did not reveal a statistically significant difference (p > 0.05). The *Forward/backward meeting point* average for all channel types was up to 33% ± 5% (n = 6 cultures). However, this feature only partially represented the real efficacy of the microchannel design; the group of the axons that followed the first axons grew much slower and contacted the boundaries of the microchannel.

Next, the neurites that originated from the *Target* chamber continued to grow in the backward direction alongside the other neurites. We estimated the length of the backward growth in the microchannel of the growing neurites until it was clearly distinguishable (Fig. 2E bottom, C). On average, the neurites reached 46% ± 9% of the microchannel length from the end of the microchannel (n = 6 cultures) (282 µm ± 54 µm) (Fig. 4A, red). Only neurites in the large BT and BZ sections grew almost to the beginning of the microchannel. Further growth was prevented by the high density of the axons that were still growing inside the microchannel from the *Source* chamber. The one-way ANOVA analysis (F-test) revealed a statistically significant difference (p < 0.05) between the observed lengths. The greatest significant difference was observed for the BT (Big triangle) type, which was different from the other six types (sS, MS, sZ, MZ, sT, MT). “Big zig-zag” (BZ) also showed a statistically significant difference with two types of the microchannels (MZ, sT). Of note, the most effective design according to that measure was MZ (medium zig-zag) (36% ± 10% *maximum backward axon growth*) (n = 6 cultures). This design should be intuitively effective because it has trap structures of the “horn” shape with a narrow angle, and there is a balance between the number of bottlenecks and the section size. Finally, we applied a two-way ANOVA (F-test) to estimate whether the size or the shape of the segment affected the maximum backward growth characteristic. We found that the size played a greater role in backward growth *(n* = *6 cultures, 138 channels, p* < *0.05)*.

Additionally, we measured the axon *growth velocity* as it passed two bottlenecks (Fig. 4B). We found that the maximum velocity of the forward growth was observed in the triangular shape of the sections with the smallest size (sT). This result can be explained by the small area inside the section, which limited the growth trajectory variability during sprouting. Moreover, the bottlenecks along the channel were aligned, which also provided faster elongation dynamics. We found that the maximum velocity of backward growth was observed in the “Zig-zag” shape of the sections of a medium size (MZ). The forward growth velocity was more than the backward growth velocity in the microchannels sT and MZ (Mann-Witney test, p < 0.05) (Fig. 4B). Note that to measure the velocity, we considered only single visible neurites and did not count the actual complex dynamics of the bundle of fibres, as illustrated in Support Video S6. We also found that the mean velocity of elongation was not significantly different for various microchannel designs (One-way ANOVA, n = 6, 121 sections, p > 0.05).

Next, we estimated the angular directions of the neurites when passing the bottleneck on the way to the *Target* chamber. We analysed two types of bottlenecks: “Prolonged” for “Zig-Zag” microchannels and “Short” for “Triangle” shaped sections. Most of the neurites continued to grow forward with a small variability in the range of 0–30 degrees and the straight forward direction was the most likely (Fig. 4D). Note that the distribution for the angles in the “triangle” sections was monotonous in contrast to the data from the other type of bottleneck (Fig. 4C). The results demonstrate that such microchannel shapes guide axons in a preferred direction towards the *Target* chamber.

### Electrophysiological activity on MEA coupled with microfluidic device

**Figure 5:**
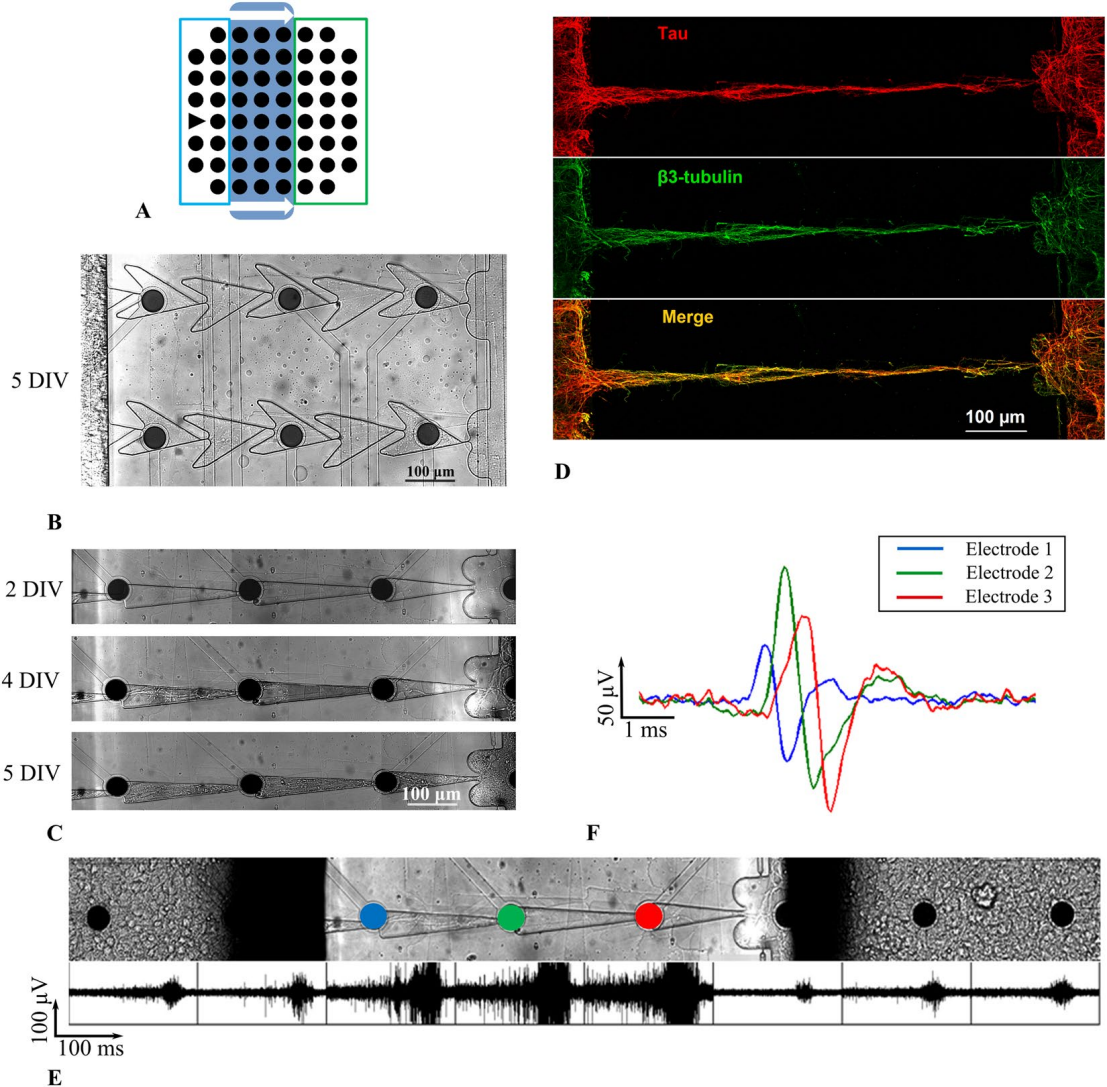
Bursting activity recorded on a multielectrode array combined with a microfluidic chip. (**A**) Schematic view of the microfluidic device mounted to the MEA. Blue box- *Source* chamber, green box- *Target* chamber, triangle- reference electrode. (**B**) Axons grew through the microchannels with 100-µm “Zig-zag” segments, 5 DIV. (**C**) Axons grew through the microchannels with narrow triangular segments up to 5 days. (**D**) Immunostaining image of the neuronal membrane (b3-tubulin, green) and axonal compartments (tau, red). The yellow colour on the merged image (bottom) indicates axons in the culture. (**E**) Electrophysiological signals recorded from the MEA electrodes. Spontaneous spiking activity consisted of 100–200 ms bursts. (**F**) Single spike propagation recorded from three electrodes inside the microchannel. Blue, red and green colours of the signals corresponded to the electrodes from a single microchannel.

The microfluidic chips were combined with MEA to study the direction of spiking activity propagation between two cultures coupled by microchannels (see Methods). We used microchannels that consisted of triangular narrow segments and “Zig-zag” microchannels with 100 mm long segments (Fig. 5A,B,C). Such designs showed minimal backward axon growth and were then considered main candidates to provide unidirectional synaptic connectivity. Note that the microchannels were aligned to the microelectrodes to record spiking activity. On 6–8 DIV, the axons completely filled the microchannels and provided connectivity between the subcultures (Fig. 5D). To classify the neurite inside the microchannels, the cultures were stained on 6 DIV with a specific antibody against axonal Tau (Fig. 5D, red) and neuronal membrane b3-tubulin (Fig. 5D, green). Axonal structures were visible in whole microchannel (Fig. 5D, yellow). Therefore, most of the neurites connecting the chambers were neuronal axons.

We recorded spiking activity from the MEAs starting from 10 DIV until 25 DIV every 5 days. We observed *Bursting activity* in both cultures in the microfluidic chip (Fig. 5E). The spike amplitudes recorded from the axons were greater (30–300 µV) than in the chambers due to high resistance (low medium volume) in the microchannels. Individual spontaneously generated spikes in the *Source* chamber propagated through the microchannels to the *Target* chamber with a velocity of 398 ± 98.8 mm/s (n = 2 cultures, 13 channels) (Fig. 5F). Similar results were shown in a study of symmetric microchannels^44^.

**Figure 6:**
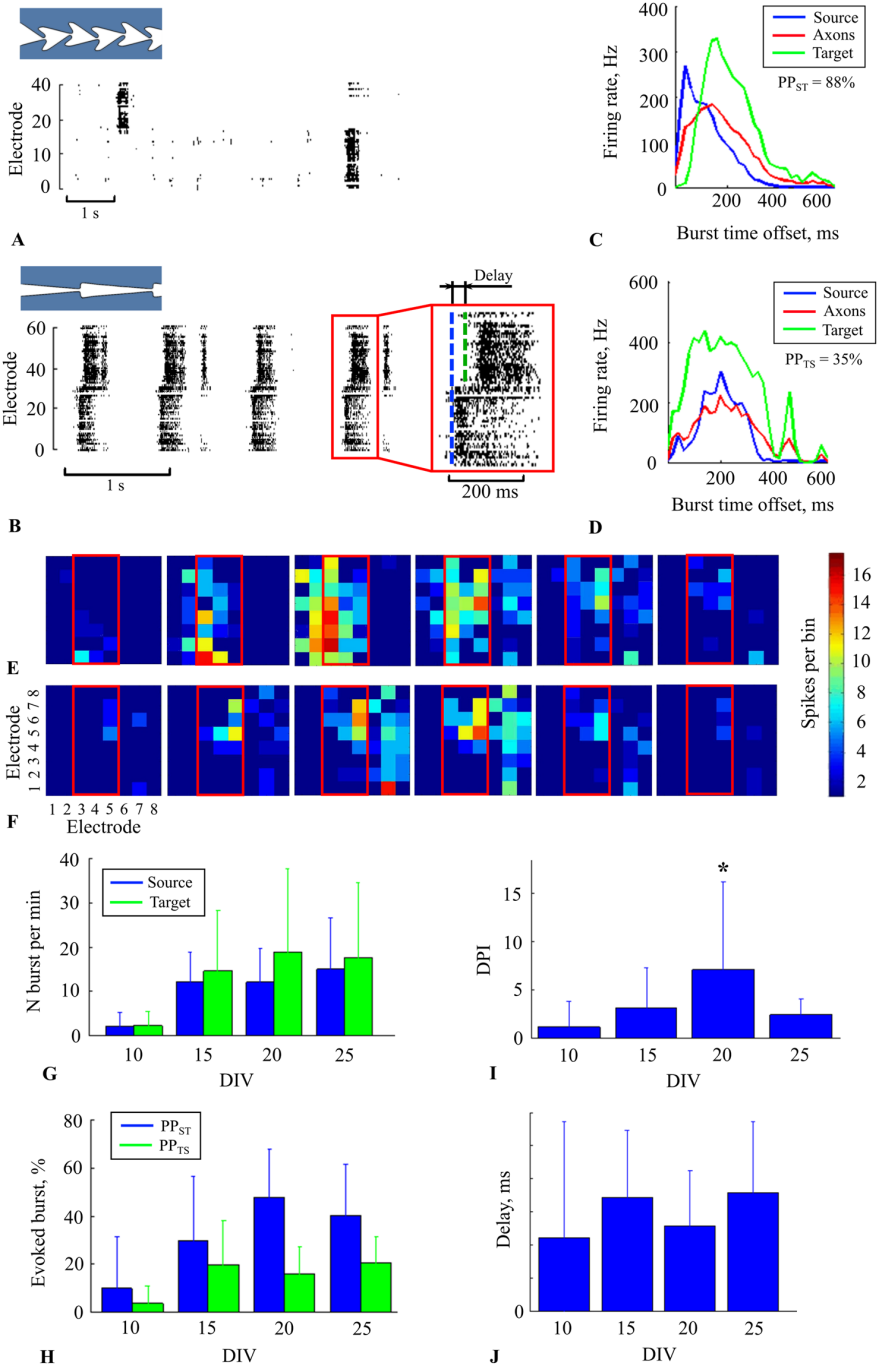
Bursting activity propagated between two cultures through the microchannels. (**A**) Raster plot of spiking activity in the chip with “Zig-zag” shaped segments with 100-mm microchannel length. Sequential activations, e.g., signal propagation, did not occur. (**B**) Raster plot of the activity in the chip with narrow “Triangle” microchannels. There was sequential activation of the chambers, e.g., bursts propagated from the *Source* to the *Target* chamber with a delay. The beginning of a burst is marked by dashed lines (blue for the *Source*, green for the *Target*). *Firing rate profiles* indicate propagated bursts (see Methods) from the *Source* electrodes through the microchannels to the *Target* electrodes (**C**) and the bursts in opposite direction (**D**). Blue, green and red lines indicate the average firing rate during the burst recorded from the all electrodes in the *Source* chamber, microchannels and the *Target* chamber, respectively. The activity was recorded on DIV 21. (**E**) Time-lapse images of the single burst propagation in the forward direction from the *Source* to the *Target* chamber. Each of 60 squares corresponded to the MEA electrodes sites, and the colour grade encoded the number of spikes within every 5 ms time bin of the spiking activity during the burst propagation. (**F**) Time-lapse images of the burst propagation that originated in the *Target* chamber but did not induce a burst in the *Source* culture. (**G**) Average number of bursts per minute in the *Source* (blue) and the *Target* (green) cultures on the MEA during culture development from 10 DIV to 25 DIV (n = 9 cultures). (**H**) Average number of bursts in the *Target* culture induced by the *Source* culture (blue), and in the *Source* culture induced by the *Target* culture (green) during development (n = 9 cultures). (**I**) *Directional propagation index* during culture development (See Methods) (n = 9 cultures). A significant difference was found on the 20th DIV. (**J**) Delay between burst beginnings in the *Source* chamber and the response burst in the *Target* chamber.

Starting from 10 DIV, we observed spontaneous bursting activity within both chambers. In 9 out of 14 cultures, we found burst propagation between the chambers through the microchannels (see Methods) (Fig. 6B). In other cultures, the bursts in the chambers were uncorrelated, but spiking activity within the microchannels was observed. Next, we estimated the fraction of the bursts generated in the *Source* chamber that then propagated through the microchannels and evoked a burst in the *Target* chamber (see Methods). The percentage of bursts propagated to the *Target* chamber (PP_S-T_) was equal to 88% (170 out of 193) on 20 DIV; raster activity is illustrated in Fig. 6B. However, the bursts also propagated in backward direction from the *Target* to the *Source* chamber. The percentage (PP_T-S_) of propagated bursts was relatively small, 35% (143 out of 409) (Fig. 6D), while the other bursts in the *Target* chamber did not induce any response bursts in the *Source* chamber. This characteristic was specific for each culture and depended on the number of axons that grew through the microchannels, which was defined by the initial plating conditions in both chambers. The average PP_S-T_ from 9 cultures on DIV 20 was 47 ± 20% and the PP_T-S_ was 15 ± 11%.

Propagation dynamics can be represented as the average *firing rate profiles* taken as the total number of spikes within each 20 ms time bin of recorded activity, which was estimated for the initiation burst in the *Source* chamber (Fig. 6C, blue), in the microchannels (Fig. 6C, red) and for the evoked bursts in the *Target* chamber (Fig. 6C, green). The spiking activity of the burst initiation, i.e., burst activation, appeared in the *Source* and in the microchannels simultaneously, while the response burst occurred after a small delay in the range of tens of milliseconds. The difference between the timing of the first spikes in the burst from two chambers (Fig. 6B, inset) determined the average synaptic delay between two cultures. In Fig. 6C,D, the difference was equal to 193 ± 142 ms. On average, the delay was 129 ± 83 ms (n = 9 cultures) on 20 DIV. We visualized the burst propagation dynamics as a sequence of 8 × 8 colour images. Each square out of 60 corresponded to the MEA electrode site and the colour grade encoded the number of spikes within every 5 ms time bin of the spiking activity during the burst. Time-lapse images of one of the propagating bursts are illustrated in Fig. 6E (see Support Video 7 for the full burst). This representation shows the directional propagation of activity between the cultures. Time-lapse images of spontaneously generated bursts in the *Target* chamber are presented in Fig. 6F (see Support Video 8 for the full burst). Bursting activity recruited the neurons in the *Target* chamber and in the microchannels, but no spikes were observed in the *Source* chamber.

Next, we quantified the microchannel efficiency of unidirectional signal transmission. We estimated the *directional propagation index* (DPI) for each culture, defined as the ratio between forward to backward propagated bursts. The DPI for the culture activity presented in Fig. 6B recorded on 20 DIV was equal to 3.8. On average, on 20 DIV, the DPI was equal to 7 ± 9 (n = 9 cultures).

Next, it was important to study changes in the functional characteristics of activity propagation during culture development from DIV 10 to DIV 25. On average, the bursting frequency in both cultures increased during the second week of development (Fig. 6G). Propagation through the microchannels also increased in the forward and backward directions with activity increase (Fig. 6H), while the forward propagation remained higher than the backward propagation. The propagation index DPI had a maximum value on the 20th DIV and was equal to 7 ± 9 (mean and SD) (Fig. 6I), which was significantly different from the measures obtained on the other days (ANOVA, p < 0.05). Note that the burst propagation delay was quite variable during the development, but the mean values did not significantly change (ANOVA, p > 0.05) and were in the range of 147 ± 120 ms (Fig. 6J).

**Figure 7:**
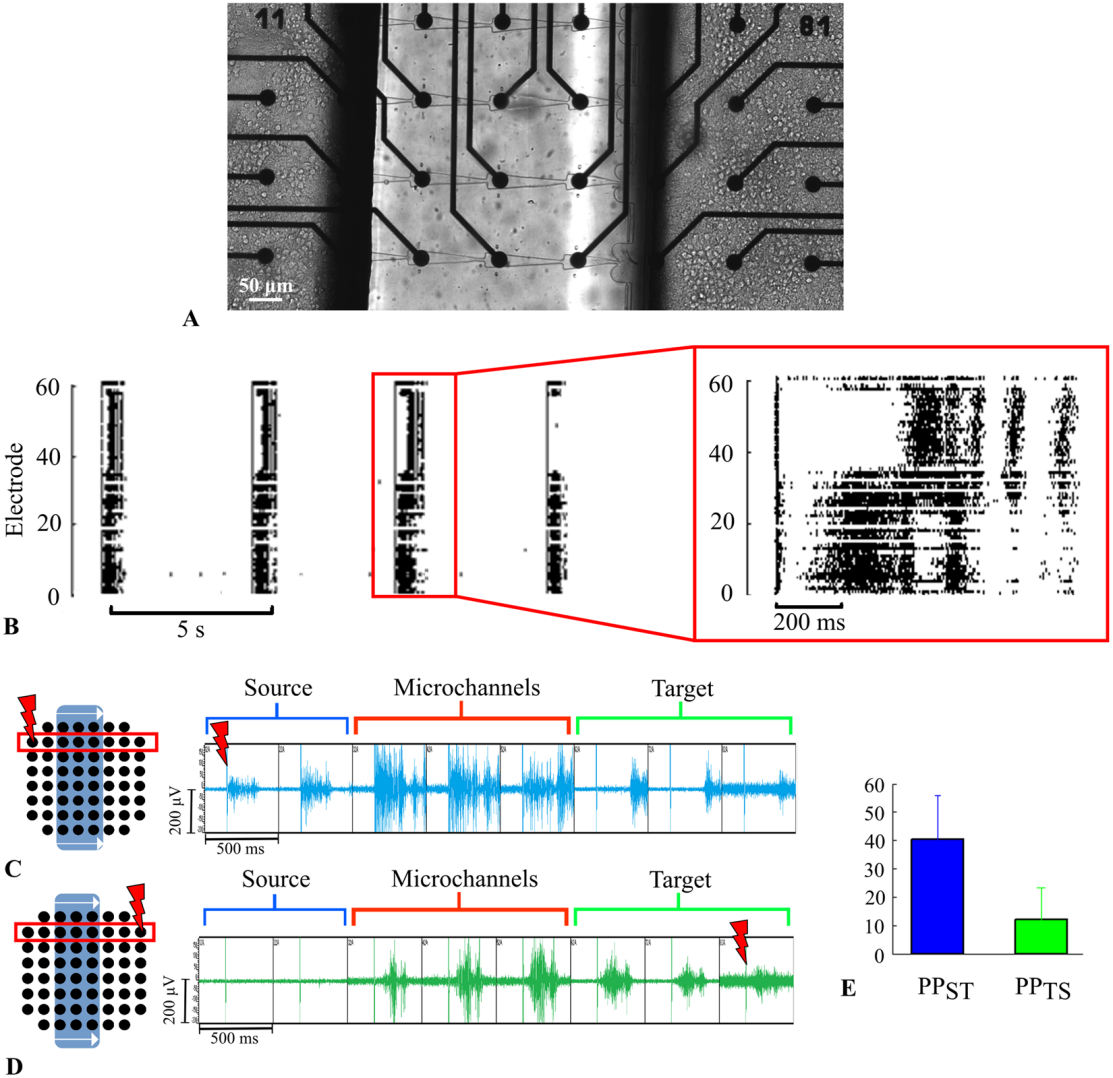
Stimulus-induced burst propagation through the microchannels. (**A**) Hippocampal neurons cultured on microfluidic chips with narrow triangle microchannels coupled with MEA. (**B**) Electrical pulses in the *Source* culture evoked bursting activity propagated to the *Target* culture with a delay. Each point on the raster plot corresponds to a spike. (**C**) Scheme of the experiment with the stimulation of the electrode in the *Source* culture (left). The raw signal of evoked burst propagation from the *Source* to the *Target* culture (right). (**D**) Scheme of the experiment with the stimulation of the electrode in the *Target* culture (left). The evoked burst propagates to the microchannel but not to the *Source* culture (right). (**E**) Average number of bursts evoked in the *Target* culture by the stimulus of the *Source* culture (blue) and evoked in the *Source* culture by the stimulus of the *Target* culture (green) (n = 4).

Next, we applied an electrical stimulus to induce bursts in the chambers to confirm burst propagation direction through the microchannels. Low-frequency stimuli were applied to an electrode chosen at random in the *Source* chamber to induce bursts (see Methods). In response to the stimulus, we observed bursting activity in the *Source* chamber, in the microchannels and in the *Target* chamber (Fig. 7C,D). The spikes in the *Source* chamber appeared simultaneously on the axons within the microchannels. Note that the bursts in the *Target* chamber appeared after a certain delay in the range of 100–300 ms in response to the initiation burst. The stimulus applied to the *Target* chamber induced spikes only in the *Target* and the microchannels (Fig. 7D). Next, we estimated the burst propagation probability induced by the stimulus from the *Source* to the *Target* chamber (PP_S-T_) and then from the *Target* to the *Source* chamber (PP_T-S_). PP_S-T_ and PP_T-S_ were 40% ± 15% and 12% ± 11%, respectively (Fig. 7E).

## Discussion

In this study, we proposed a solution to the design of multi-layered structures of neuronal networks with directed connectivity that resembled networks in the living brain. The solution is based on a microfluidic microelectrode device composed of several chambers connected by specifically shaped microchannels that provide unidirectional axon growth and direct the transmission of electrical activity. The device was tested with primary dissociated cultures of hippocampal neurons plated separately in different chambers. We proposed the use of ten different types of microchannels (Fig. 1) of various shapes and sizes. Neurite growth was analysed with microscope images (1 frame per 20 min) taken during the first 10 days of cultured network development. We found that the neurites primarily grew in the desired direction from the *Source* to the *Target* chamber and filled the microchannels at 6–8 DIV (Fig. 3). The neurites also grew in the reverse direction from the *Target* chamber and filled the trapping segment at the end of the microchannels (Fig. 2E). We found, however, that a single trap was ultimately “safe”, and some axons could overcome “trap” shapes and change growth direction by turning the growth angle up to 180 degrees (Support Videos S9, S10). In such cases, several trapping segments were needed to decrease the reverse growth probability. Note that compared to a similar approach of controlling the direction of the neural network via a micropatterning method^28^, our method provides a clear separation of cell somas from axons and various types of cells can be co-cultured to study complex and realistic networks.

The functional efficiency of the microchannels was confirmed via the electrophysiology analysis of the MEA recordings. The microfluidic chip was coupled with the MEA to record the spiking activity of the neurons. The amplitude of spiking activity observed in the microchannels was greater (30–300 mV) than the activity in the chambers due to the high resistance of the medium around the recording area. A similar effect was observed and described in several earlier studies^39,45,46^. To maximize the amplification effect, we placed the bottleneck of the microchannels on the electrodes (Fig. 5C). The velocity of the spike propagation was 398 ± 98.8 mm/s (n = 2 cultures, 13 channels), which agrees with other studies of axons grown through symmetric microchannels^39,47–49^.

Spontaneously generated bursting activity in the *Source* chamber always induced spiking activity within the microchannels, and a certain fraction of the bursts evoked response bursting in the *Target* chamber (Fig. 6). Such directional burst propagation was observed preferably in the narrow triangular shaped microchannels (Fig. 6,B). This neural network structure permits the separated localization of presynaptic (*Source* chamber) and postsynaptic cells (Target chamber). Microelectrodes placed inside and near the end (in the *Target* chamber) of the microchannel can be used for the electrical stimulation of pre- and postsynaptic cells to study synaptic plasticity effects on a network level.

The delay between the bursts propagated through the microchannels did not significantly change and was equal to 147 ± 120 ms (mean ± SD) from 10 DIV to 25 DIV (Fig. 6J). Similar results were obtained in two cultures coupled by symmetric microchannels with bi-directional bursting activity propagation^15,17,38^. We suggest that the difference between such high delays and synaptic transmission delays (~5 ms) can be explained by a certain threshold of fraction of the neurons in the *Target* culture that should be depolarized by external activity to induce evoked bursts.

Such stable connectivity can be used in the study of homeostatic and long-term synaptic plasticity in various conditions, such as in models of neurodegenerative diseases or in the presence of neuromodulators. The microfluidic devices integrated with MEA can be effectively used in the research on neural network synaptic architecture development^15,50^, network-wide synaptic plasticity and the interaction between various types of neurons^2^. Such devices can also host a network of living neurons that implement logic functions and basic operations^30^.

In conclusion, we note that our two-chamber device can be easily expanded to a system composed of several inter-connected chambers with different morphologies generated from a 3D design. Therefore, more complex neuronal circuitries, such as a closed looped network that mimics interconnected EC, DG, CA1 CA3 brain networks, can be developed. We believe that the further development of microchannel techniques will be useful in the design of new scaffold structures with integrated neuronal networks for neurotransplantation *in vivo*.

## Electronic supplementary material

Video S1

Video S2

Video S3

Video S4

Video S5

Video S6

Video S7

Video S8

Video S9

Video S10

Supplementary Information

Video legends
