# Supplementary material for: Design of Cultured Neuron Networks in vitro with Predefined Connectivity Using Asymmetric Microfluidic Channels
Source: Sci Rep. 2017 Nov 15;7:15625. doi: 10.1038/s41598-017-15506-2 (PMC5688062; doi:10.1038/s41598-017-15506-2)
Supplement: Supplementary file 11 — Supplementary Information [file 41598_2017_15506_MOESM11_ESM.pdf]

Design of Cultured Neuron Networks *in vitro* with Predefined Connectivity Using Asymmetric Microfluidic Channels

Gladkov Arseniy[1,2,*], Pigareva Yana[1], Kutyina Daria[1], Kolpakov Vladimir[1], Bukatin Anton[3], Mukhina Irina[1,2], Kazantsev Victor[1], Pimashkin Alexey[1]

[1] Lobachevsky State University of Nizhny Novgorod, Laboratory of Neuroengineering, Nizhny Novgorod, 603950, Russia
[2] Nizhny Novgorod State Medical Academy, Central Research Laboratory, Nizhny Novgorod, 603950, Russia
[3] Saint-Petersburg National Research Academic University of the RAS, Laboratory of Nanobiotechnology, Saint-Petersburg, 194021, Russia,

* gladkov@neuro.nnov.ru

Supplementary information

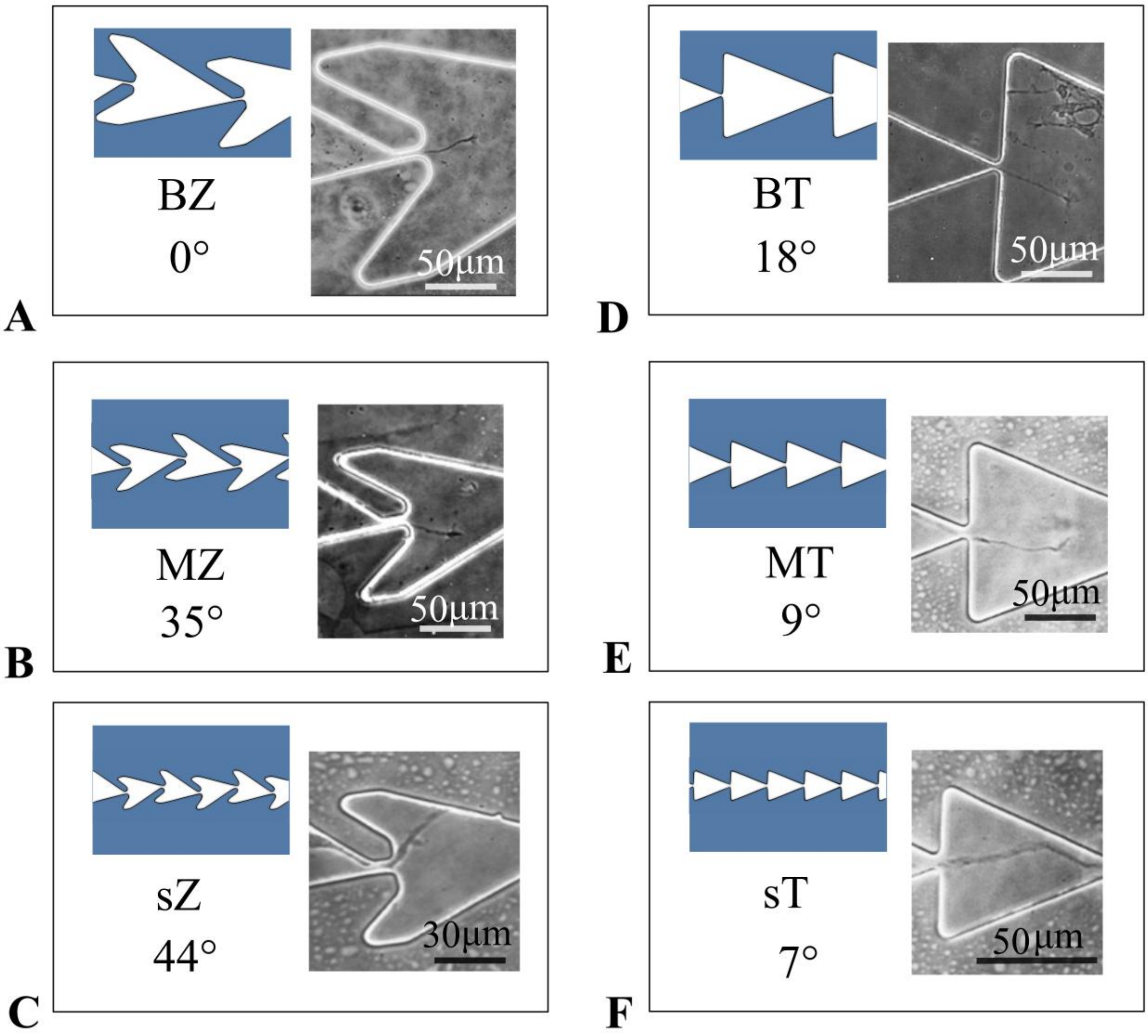

Support figure S1. Angle of the axon growth trajectory when it passed the bottleneck in different types of microchannels. (**A**) Big microchannel with "Zig-zag" segment. (**B**) Medium microchannel with "Zig-zag" segment. (**C**) Small microchannel with "Zig-zag" segment. (**D**) Big microchannel with "Triangle" segment. (**E**) Medium microchannel with "Triangle" segment. (**F**) Small microchannel with "Triangle" segment
