# Supplementary material for: Design of Cultured Neuron Networks in vitro with Predefined Connectivity Using Asymmetric Microfluidic Channels
Source: Sci Rep. 2017 Nov 15;7:15625. doi: 10.1038/s41598-017-15506-2 (PMC5688062; doi:10.1038/s41598-017-15506-2)
Supplement: Supplementary file 12 — Video legends [file 41598_2017_15506_MOESM12_ESM.pdf]

Design of Cultured Neuron Networks *in vitro* with Predefined Connectivity Using Asymmetric Microfluidic Channels

Gladkov Arseniy[1,2,*], Pigareva Yana[1], Kutyina Daria[1], Kolpakov Vladimir[1], Bukatin Anton[3], Mukhina Irina[1,2], Kazantsev Victor[1], Pimashkin Alexey[1]

[1] Lobachevsky State University of Nizhny Novgorod, Laboratory of Neuroengineering, Nizhny Novgorod, 603950, Russia
[2] Nizhny Novgorod State Medical Academy, Central Research Laboratory, Nizhny Novgorod, 603950, Russia
[3] Saint-Petersburg National Research Academic University of the RAS, Laboratory of Nanobiotechnology, Saint-Petersburg, 194021, Russia,

* gladkov@neuro.nnov.ru

Video Legends

S1. An example of neurite growth in a microchannel composed of medium “Zig-zag” shaped segments (100 µm in length).
S2. An example of neurite growth in a microchannel composed of large “Zig-zag” shaped segments (200 µm in length).
S3. An example of neurite growth in a microchannel composed of “triangle” shaped segments (66 µm in length).
S4. Neurites growing “backward” from the *Target* culture alongside the neurite grown from the *Source*.
S5. An example of neurite growth in a microchannel composed of “Spine” shaped segments (100 µm in length).
S6. Visible single neurites and actual complex dynamics of a bundle of fibres in a microchannel composed of “triangle” shaped segments (100 µm in length).
S7. Time-lapse images of one of the propagating bursts from the *Source* chamber to the *Target*. Each square corresponds to the MEA electrode site and the colour grade encodes the number of spikes within every 5 ms time bin of the spiking activity during the burst.
S8. Bursting activity recruited the neurons in the *Target* chamber and in the microchannels, but no spikes were observed in the *Source* chamber. Each square corresponds to the MEA electrode site and the colour grade encodes the number of spikes within every 5 ms time bin of the spiking activity during the burst.
S9. An example of an axon that changes the growth direction and turns its growth angle up to 180 degrees in a large size “Zig-zag” shaped segment (200 µm in length).
S10. An example of an axon that overcomes “trap” shapes and changes growth direction by turning its growth angle up to 180 degrees in medium size “Zig-zag” shaped segments (100 µm in length).
